# Supplementary material for: Physiologic recovery of Mycobacterium tuberculosis from drug injury: A molecular study of post antibiotic effect in vitro and in vivo
Source: PLoS Pathog. 2026 Apr 22;22(4):e1014131. doi: 10.1371/journal.ppat.1014131 (PMC13128115; doi:10.1371/journal.ppat.1014131)
Supplement: S1 File — (DOCX) [file ppat.1014131.s001.docx]

**SUPPLEMENTAL INFORMATION**

**Physiologic recovery of *Mycobacterium tuberculosis* from drug injury: A molecular study of post antibiotic effect *in vitro* and *in vivo***

Jo Hendrix, Reem Al Mubarak, Matthew J. Reichlen, Samuel T. Tabor, Adeline Bateman, Lisa M. Massoudi, Karen Rossmassler, Firat Kaya, Mathew D. Zimmerman, Holly Nielsen, Elizabeth A. Wynn, Martin I. Voskuil, Gregory T. Robertson, Camille M. Moore, Nicholas D. Walter

Table of Contents

[1. Supplemental Methods 3](#_Toc226127999)

[1.1 Sampling for in vitro experiment 3](#_Toc226128000)

[1.2. Table A. Drug dosages 4](#_Toc226128001)

[1.3. Bacterial bioinformatics 4](#_Toc226128002)

[1.4. Table B. Curated gene categories 5](#_Toc226128003)

[2. Supplemental Results 8](#_Toc226128004)

[2.1. Table C. Gene set enrichment of hierarchical gene clustering 8](#_Toc226128005)

[2.2. Transcriptional change of select gene sets during recovery in axenic culture 10](#_Toc226128006)

[2.3. Table D. Toxin gene clustering in axenic culture 11](#_Toc226128007)

[2.4. Comparison of transcriptome at end of treatment compared to pre-treatment control in murine samples 12](#_Toc226128008)

[2.5. Concordance of effect of treatment on expression with previous study 13](#_Toc226128009)

[2.6. Enrichment of Clustering in gene differentially expressed by treatment in mice 14](#_Toc226128010)

[2.7. Comparison of CFU and RS ratio to baseline 15](#_Toc226128011)

[2.8. Transcriptional change of select gene sets during recovery in mice 17](#_Toc226128012)

[2.9. Table J. Toxin gene clustering in mouse samples 18](#_Toc226128013)

[3. References 19](#_Toc226128014)

# 1. Supplemental Methods

## 1.1 Sampling for *in vitro* experiment

After the washout step, cultures were diluted to an OD of 0.05 then allowed to grow until exceeding an OD_600_ of 2 or 15 days. Cultures were frequently sampled during this time to gather data on the OD_600_, CFU, RS ratio, and for SEARCH-TB sequencing. Exact times that samples were extracted are displayed here.

**Fig A. Sampling schematic of in vitro study.** Timeline of samples collected in the (a) untreated control arm, (b) 6-hour treatment group, and (c) 48-hour treatment group after washout step. A dot indicates that a give metric was collected for four samples at the specified timepoint for that group.

## 1.2. Table A. Drug dosages

**Table A**. Drugs and doses used in treatment with the HRZE regimen in murine samples.

| **Drug** | **Dosage** | **Abbreviation** |
| --- | --- | --- |
| Isoniazid | 10m/kg | H |
| Rifampin | 10mg/kg | R |
| Pyrazinamide | 150mg/kg | Z |
| Ethambutol | 100mg/kc | E |

## 1.3. Bacterial bioinformatics

After sequencing, bacterial reads were trimmed by Skewer v0.2.2^1^ using an adapter sequence of 5’- CTGTCTCTTATACACATCT-3’, a length of 50-175 bases, and an end quality of 20. Individual reads were reunited with their mate pair using PairFQ lite v0.17.0.^2^ Paired-end reads were then aligned to the 2016 *Mtb* Erdman reference assembly using Bowtie2 v2.5.0^3^ with default parameters. Mapped sequences were counted using HtSeq v1.0^4^ with the default parameters.

The SEARCH-TB panel amplifies *Mtb* 3,568 genes. In this experiment, 12 genes had high incidences of 0 expression values, so our analysis included 3,552 genes.

## 1.4. Table B. Curated gene categories

**Table B**. Curated gene categories used for average expression analysis

Gene categories curated from the literature were used in category enrichment analysis. Along with the reference, the total number of genes from each source along with the number of genes from each source which were in the SEARCH-TB assay is given. Other gene sets used for enrichment analysis are available in Cole *et al.*, 1998.^5^

| **Category** | **Genes Total** | **Genes In Assay** | **Source** |
| --- | --- | --- | --- |
| ABC transporters | 72 | 68 | ^6^ |
| ABC transporters - Anion Transporters | 7 | 7 | ^7^ |
| ABC transporters - Metal Transporters | 5 | 5 | ^7^ |
| ABC transporters - Type I peptide and amino acids | 8 | 6 | ^7^ |
| ABC transporters - Type I phosphate | 8 | 8 | ^7^ |
| ABC transporters - Type I Sugar Importers | 12 | 12 | ^7^ |
| Alternative ribosomal proteins | 5 | 4 | ^8^ |
| Antigen 85 | 3 | 3 | ^9^ |
| Antitoxins | 76 | 72 | ^10^ |
| Arabinogalactan (AG) | 20 | 19 | ^11^ |
| Beta Oxidation | 18 | 17 | ^12^ |
| Cell wall synthesis | 40 | 40 | ^13^ |
| Cholesterol A and B ring degradation | 10 | 10 | ^14^ |
| Cholesterol C and D ring degradation | 5 | 5 | ^14^ |
| Cholesterol side chain degradation | 33 | 33 | ^14^ |
| Cutinase-Like Protein (CULP) | 7 | 7 | ^15^ |
| Cytochrome *bcc/aa3* supercomplex | 7 | 6 | ^16^ |
| Cytochrome *bd* oxidase | 4 | 3 | ^16^ |
| Diacyltrehalose (DAT) and Pentaacyltrehalose (PAT) | 5 | 5 | ^17^ |
| DNA replication and repair | 27 | 25 | ^18^ |
| DosR | 48 | 48 | ^19^ |
| Efflux Pumps and Transports | 26 | 25 | ^20^ |
| Enduring Hypoxic Response | 161 | 149 | ^21^ |
| Esterases (Lip family) | 22 | 20 | ^15^ |
| ESX1 | 19 | 18 | ^22^ |
| ESX2 | 12 | 12 | ^22^ |
| ESX3 | 11 | 9 | ^22^ |
| ESX4 | 7 | 7 | ^22^ |
| ESX5 | 15 | 11 | ^22^ |
| Fatty Acid Synthases I | 1 | 1 | ^5^ |
| Fatty Acid Synthases II | 10 | 9 | ^23^ |
| Fumarate reductase | 4 | 4 | ^5^ |
| Kas operon | 5 | 3 | ^24^ |
| kstR1 regulon | 74 | 70 | ^25^ |
| kstR2 regulon | 15 | 14 | ^25^ |
| LAM | 15 | 14 | ^26^ |
| LpqY-SugA-SugB-Sug trehalose transporter | 5 | 5 | ^7^ |
| Mce1 | 7 | 7 | ^5^ |
| Mce2 | 7 | 6 | ^5^ |
| Mce3 | 7 | 7 | ^5^ |
| Mce4 | 7 | 7 | ^5^ |
| mmpL | 14 | 14 | ^27^ |
| mmpS | 5 | 5 | ^28^ |
| Mycobactin Biogenesis | 10 | 10 | ^29^ |
| Mycolic Acid Modification | 12 | 12 | ^30^ |
| Mycolic Acid Transfer and Modification | 7 | 7 | ^30^ |
| NADH dehydrogenase type I | 14 | 12 | ^31^ |
| NADH dehydrogenase type II | 2 | 2 | ^31^ |
| Nitrate import and reductase | 6 | 8 | ^5^ |
| Oxidative Stress | 51 | 50 | ^32^ |
| Phthiocerol Dimycocerosate (PDIM) | 20 | 20 | ^33^ |
| Peptidoglycan (PG) | 34 | 32 | ^34^ |
| PG synthesis1- cytoplasmic steps | 7 | 7 | ^34^ |
| PG synthesis2- membrane-associated steps | 3 | 3 | ^34^ |
| Peptidoglycan (PG) Linking | 13 | 13 | ^34^ |
| PG synthesis4- remodeling and degradation | 2 | 2 | ^34^ |
| Peptidoglycan (PG) Transport and recycling | 3 | 3 | ^34^ |
| Phospholipases C | 4 | 4 | ^35^ |
| Primary Ribosomal Protein | 53 | 50 | ^5^ |
| response to acid stress | 11 | 9 |  |
| Ribosomal hibernation | 4 | 4 | ^36^ |
| Sigma Factors | 13 | 12 | ^6^ |
| Stringent Response - Induced | 70 | 58 | ^37^ |
| Stringent Response - Repressed | 78 | 66 | ^37^ |
| Succinate Dehydrogenase Types I and II | 7 | 7 | ^38^ |
| Toxin-Antitoxin | 152 | 143 | ^10^ |
| Toxins | 76 | 74 | ^10^ |
| Transcription Factors | 198 | 187 | ^6^ |
| Trehalose | 5 | 5 | ^39^ |
| Triacylglycerol Synthases | 16 | 14 | ^40^ |
| UgpABCE glycerophosphocholine transporter | 4 | 4 | ^7^ |
| Universal Stress Proteins | 10 | 9 | ^6^ |
| WhiB-Like transcription factors | 7 | 7 | ^41^ |
| Zur regulon | 20 | 17 | ^42^ |

* Primary Ribosomal Proteins include ribosomal proteins identified by Cole *et al.* but exclude the four alternative ribosomal proteins

# 2. Supplemental Results

## 2.1. Table C. Gene set enrichment of hierarchical gene clustering

Hierarchical clustering was performed on genes differentially expressed after 6 and 48-hours of treatment compared to pre-treatment, identifying four broad categories. Clusters 1 and 2 decreased in expression over treatment time while Clusters 3 and 4 increased in expression over treatment time. For enrichment analysis, Clusters 1 and 2 were combined to identify gene sets that were overrepresented in genes that are suppressed by treatment. Similarly, Clusters 3 and 4 were combined to identify gene sets that were overrepresented in genes that were activated by treatment. In Fig 1h-I in the main text, only the top ten enrichments were displayed. The below table lists all the significant enrichments with adjusted p-value < 0.05 after BH correction.

**Table C.** Significant gene set enrichments for clustering of genes during PAE phase.

| Category | #significant genes | # genes in category | Adj-P | Cluster |
| --- | --- | --- | --- | --- |
| Aerobic | 24 | 28 | 0.00012 | Cluster 1&2 Combined |
| Antitoxins | 44 | 70 | 0.0052 | Cluster 1&2 Combined |
| ATP-proton motive force | 8 | 8 | 0.011 | Cluster 1&2 Combined |
| Branched amino acid family | 10 | 12 | 0.035 | Cluster 1&2 Combined |
| Chaperones Heat shock | 12 | 14 | 0.012 | Cluster 1&2 Combined |
| Drug targets | 28 | 36 | 0.00038 | Cluster 1&2 Combined |
| Energy Metabolism | 137 | 266 | 0.013 | Cluster 1&2 Combined |
| ESX1 | 17 | 18 | 0.00016 | Cluster 1&2 Combined |
| ESX3 | 9 | 9 | 0.0052 | Cluster 1&2 Combined |
| Lipid Biosynthesis | 39 | 58 | 0.0018 | Cluster 1&2 Combined |
| Mce1 | 7 | 7 | 0.02 | Cluster 1&2 Combined |
| Mycolic acid condensation and transfer | 7 | 7 | 0.02 | Cluster 1&2 Combined |
| NADH dehydrogenase type I | 12 | 12 | 0.00069 | Cluster 1&2 Combined |
| PDIM | 17 | 20 | 0.0018 | Cluster 1&2 Combined |
| Primary ribosomal proteins | 48 | 50 | 3.40E-14 | Cluster 1&2 Combined |
| Protein and peptide secretion | 13 | 14 | 0.0018 | Cluster 1&2 Combined |
| Protein translation and modification | 12 | 15 | 0.027 | Cluster 1&2 Combined |
| Proteins peptides and glycopeptides | 21 | 32 | 0.049 | Cluster 1&2 Combined |
| Ribosomal protein synthesis and modification | 48 | 54 | 5.80E-11 | Cluster 1&2 Combined |
| Stringent Response - Repressed | 60 | 66 | 1.70E-14 | Cluster 1&2 Combined |
| Succinate dehydrogenase I and II | 7 | 7 | 0.02 | Cluster 1&2 Combined |
| Synthesis and modification of macromolecules | 116 | 202 | 0.00022 | Cluster 1&2 Combined |
| TCA cycle | 16 | 19 | 0.0031 | Cluster 1&2 Combined |
| ABC transporters - Type I Sugar Import | 12 | 12 | 0.0024 | Cluster 3&4 Combined |
| binding proteins | 77 | 116 | 0.00023 | Cluster 3&4 Combined |
| Carbohydrates organic acids and alcohols | 18 | 19 | 0.00037 | Cluster 3&4 Combined |
| DosR | 38 | 48 | 0.00023 | Cluster 3&4 Combined |
| IS elements Repeated sequences and Phage | 43 | 60 | 0.0012 | Cluster 3&4 Combined |

## 2.2. Transcriptional change of select gene sets during recovery in axenic culture

**Fig B. Recovery of various processes in axenic culture.** (a-p) Average of batch adjusted, VST-normalized, scaled gene expression in each treatment group over time for genes involved in (a) the TCA cycle, (b) NADH dehydrogenase Type I, (c) NADH dehydrogenase Type II, (d) Succinate dehydrogenase Type I and II, (e) ATPase, (f) aerobic respiration, (g) glycolysis, (h) glyoxylate bypass, (i) pentose phosphate pathway, (j) pyruvate dehydrogenase, (k) primary ribosomal proteins, (l) Antigen 85, (m) arabinogalactan synthesis, (n) fatty acid synthesis, (o) mycolic acid synthesis, and (p) peptidoglycan synthesis. Each point represents an individual sample, and the lines connect the mean for each time point. Values are centered around the average value for the pre-treated samples so that points above and below zero represent upregulation and downregulation relative to pre-treated, respectively. (q) Hierarchical clustering of toxin genes (N=73) during the PAE phase. The heatmap shows the batch-adjusted, VST-normalized, scaled gene expression averaged across samples.

## 2.3. Table D. Toxin gene clustering in axenic culture

**Table D.** List of toxin genes sorted into each cluster in axenic culture

| Cluster | Included Toxins |
| --- | --- |
| Cluster 1 | Rv0299, Rv0598c, Rv0659c, Rv0910, Rv1114, Rv1495, Rv1546, Rv1741, Rv1962c, Rv1982c, Rv2494, Rv2527, Rv2530c, Rv3320c |
| Cluster 2 | Rv0059, Rv0240, Rv0595c, Rv0627, Rv0656c, Rv0661c, Rv0665, Rv0836c, Rv0919, Rv1242, Rv1246c, Rv1561, Rv1720c, Rv1838c, Rv1953, Rv1959c, Rv1989c, Rv1991c, Rv2019, Rv2103c, Rv2142c, Rv2231A, Rv2546, Rv2548, Rv2653c, Rv2757c, Rv2759c, Rv2863, Rv2866, Rv2872, Rv3180c, Rv3182, Rv3189, Rv3358, Rv3384c, Rv3749c |
| Cluster 3 | Rv0065, Rv0301, Rv0549c, Rv0582, Rv0609, Rv0617, Rv0624, Rv0749, Rv0960, Rv1102c, Rv1397c, Rv1942c, Rv1955, Rv2010, Rv2022c, Rv2063A, Rv2549c, Rv2596, Rv2602, Rv2697c, Rv2801c, Rv2829c, Rv3408 |

## 2.4. Comparison of transcriptome at end of treatment compared to pre-treatment control in murine samples

Treatment with HRZE for two and four weeks massively transformed the *Mtb* transcriptome, significantly altering expression of 70.3% (2497/3552) and 73.7% (2617/3552) genes relative to pre-treatment control, respectively.

**a b**


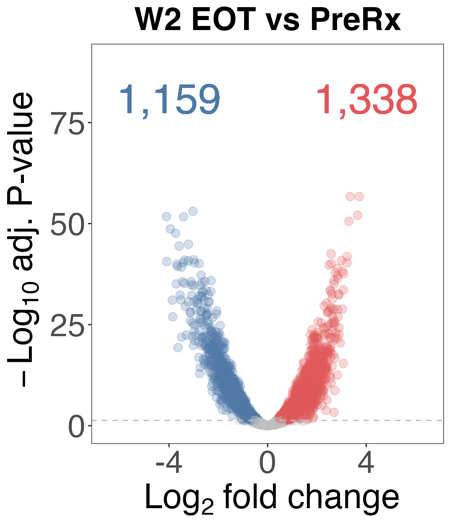

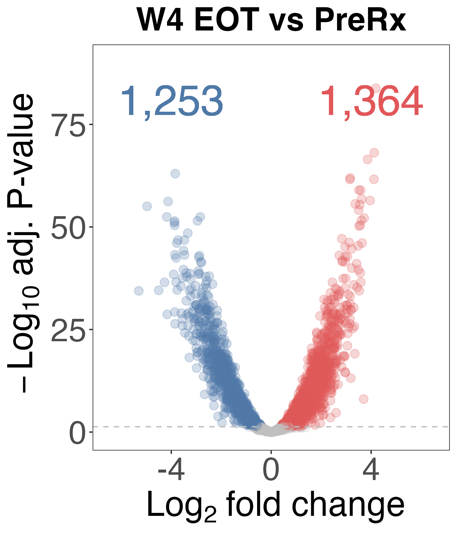


**Fig C. Comparison of transcriptome at end of treatment compared to pre-treatment control.** Volcano plot showing log_2_ fold changes and −log_10_ P-values of genes differentially expressed at the end of a two-week (a) or four-week (b) treatment compared to pre-treatment. Genes significantly down- and up-regulated relative to control (adj. P < 0.05) are shown in blue and red, respectively.

## 2.5. Concordance of effect of treatment on expression with previous study

Gene expression changes after two and four weeks of treatment were compared to previous results published by Wynn *et al.*^43^ The log_2_ fold change at the end of treatment compared to the pre-treatment (PreRx) control were determined for both studies and plotted against each other for two and four weeks of treatment. Expression changes of most genes were in the same direction, though log_2_ fold change in Wynn *et al.* tended to be smaller.

a b


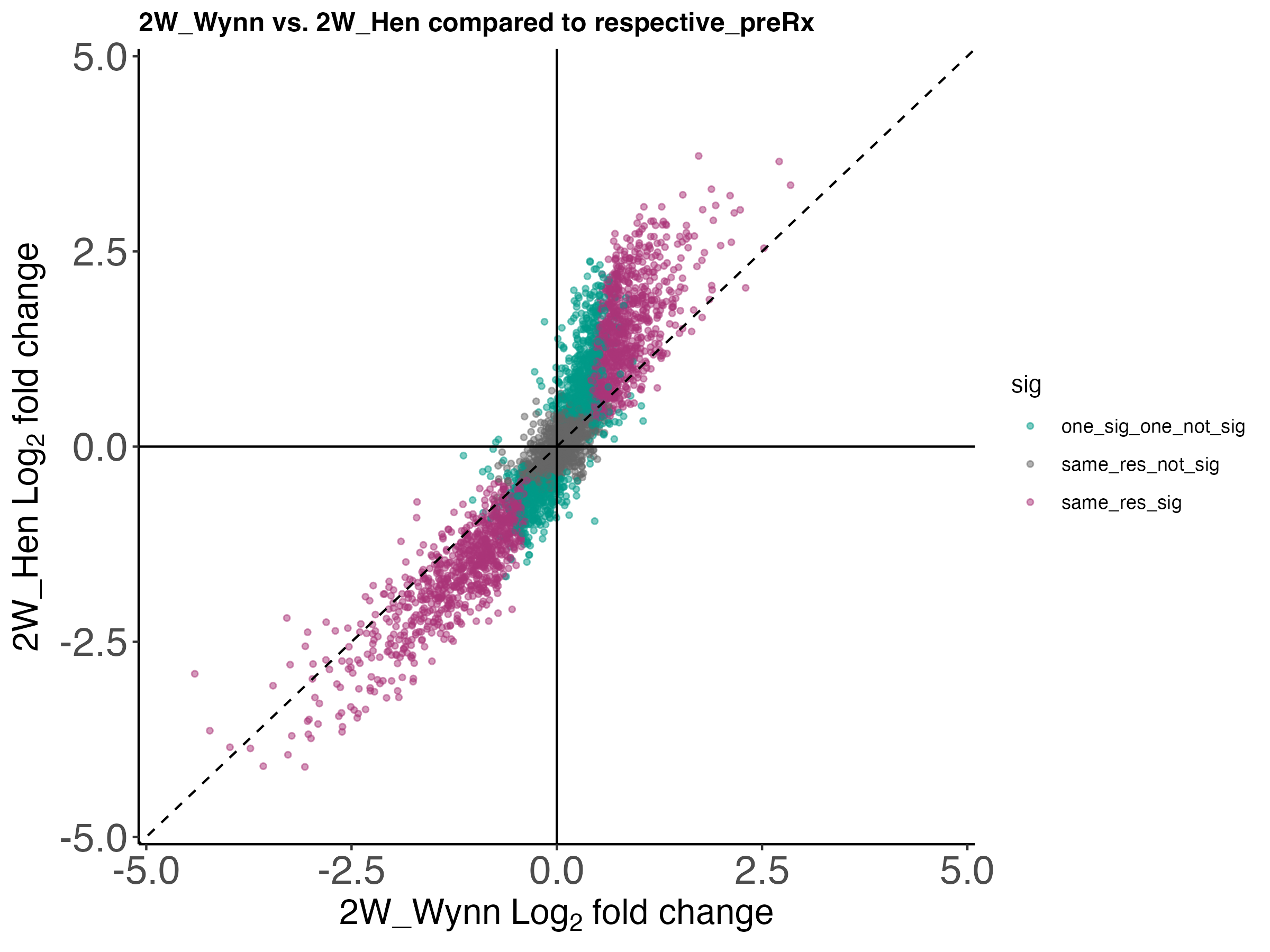

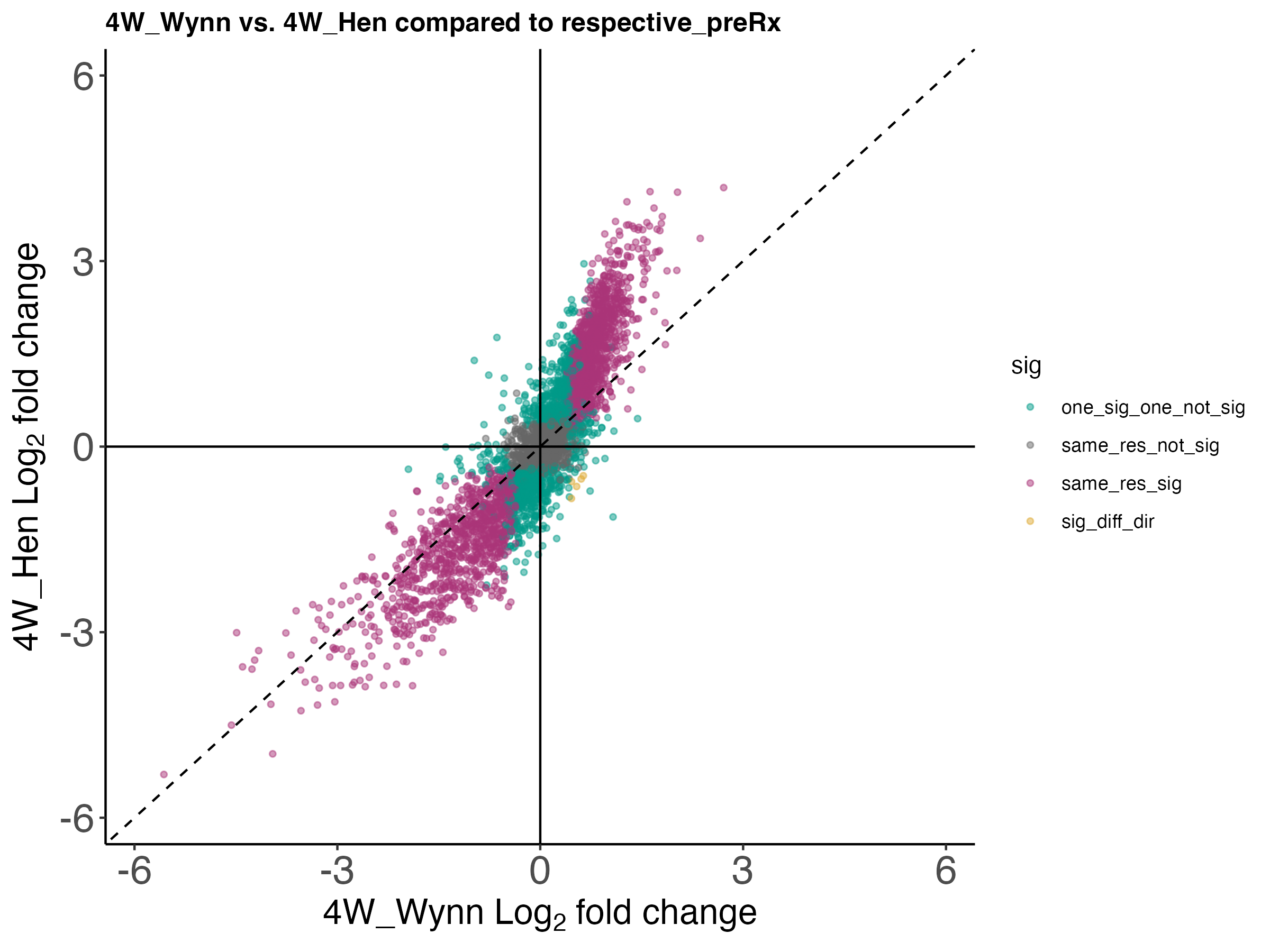


**Fig D. Concordance of effect of treatment on expression with previous study.** Comparison of two (A) or four (B) weeks of HRZE treatment versus control fold changes from this study versus Wynn *et al.* Purple shading indicates genes with concordant fold-change direction and significance between current study and Wynn *et al.* Green shading indicates genes that were significant in current or Wynn *et al.* results but not both. Gold shading indicates genes that were significant for both studies but in opposite directions. Gray shading indicates genes that were not significantly differentially expressed in either study.

## 2.6. Enrichment of Clustering in gene differentially expressed by treatment in mice

Genes that were differentially expressed after 2-week and 4-week treatment compared to the pre-treatment control were identified and clustered into three broad groups. Gene set enrichment analysis was then performed on each gene cluster. Note that there were no significant gene enrichments in cluster 2.

**Table E.** Enrichments in genes altered by treatment in mice

| Category | #significant genes | # genes in category | Adj-P | Cluster |
| --- | --- | --- | --- | --- |
| ABC transporters Type I phosphate | 7 | 8 | 0.02 | Cluster 1 |
| aerobic | 23 | 28 | 1.20E-05 | Cluster 1 |
| Amino acid biosynthesis | 49 | 82 | 9.90E-05 | Cluster 1 |
| ATP-proton motive force | 8 | 8 | 0.0026 | Cluster 1 |
| Beta Oxidation | 14 | 18 | 0.0028 | Cluster 1 |
| Cell wall synthesis | 26 | 40 | 0.0017 | Cluster 1 |
| Cholesterol A and B ring degradation | 8 | 10 | 0.026 | Cluster 1 |
| Cholesterol side chain degradation | 22 | 33 | 0.0026 | Cluster 1 |
| Cytochrome oxidase bccaa3 | 6 | 6 | 0.012 | Cluster 1 |
| DosR | 30 | 48 | 0.0016 | Cluster 1 |
| Drug targets | 23 | 36 | 0.004 | Cluster 1 |
| Energy Metabolism | 135 | 266 | 1.80E-06 | Cluster 1 |
| ESX3 | 9 | 9 | 0.0015 | Cluster 1 |
| Fatty Acid Synthases II | 7 | 8 | 0.02 | Cluster 1 |
| Fatty acids | 54 | 110 | 0.012 | Cluster 1 |
| Glycolysis | 9 | 11 | 0.013 | Cluster 1 |
| KstR1 regulon | 38 | 70 | 0.0061 | Cluster 1 |
| KstR2 regulon | 11 | 14 | 0.0083 | Cluster 1 |
| Lipid Biosynthesis | 30 | 58 | 0.034 | Cluster 1 |
| Mycolic acid condensation and transfer | 7 | 7 | 0.0055 | Cluster 1 |
| NADH dehydrogenase type I | 10 | 12 | 0.0066 | Cluster 1 |
| PDIM | 19 | 20 | 1.30E-06 | Cluster 1 |
| PG synthesis3- linkage in mature PG | 10 | 13 | 0.016 | Cluster 1 |
| Polysaccharides lipopolysaccharides and phospholipids | 7 | 8 | 0.02 | Cluster 1 |
| Primary ribosomal proteins | 47 | 50 | 2.90E-16 | Cluster 1 |
| Protein and peptide secretion | 12 | 14 | 0.0019 | Cluster 1 |
| Protein translation and modification | 12 | 15 | 0.0046 | Cluster 1 |
| Pyruvate dehydrogenase | 5 | 5 | 0.027 | Cluster 1 |
| Ribosomal protein synthesis and modification | 48 | 54 | 1.30E-14 | Cluster 1 |
| Stringent Response - Repressed | 57 | 66 | 5.30E-16 | Cluster 1 |
| Succinate dehydrogenase I and II | 7 | 7 | 0.0055 | Cluster 1 |
| Synthesis and modification of macromolecules | 96 | 202 | 0.0019 | Cluster 1 |
| TCA cycle | 16 | 19 | 0.00032 | Cluster 1 |
| Zur regulon | 12 | 17 | 0.019 | Cluster 1 |
| ABC transporters - Type I Sugar Import | 11 | 12 | 0.0011 | Cluster 3 |
| Carbohydrates organic acids and alcohols | 15 | 19 | 0.0011 | Cluster 3 |
| Esterases and lipases | 16 | 24 | 0.0085 | Cluster 3 |
| Mce3 | 7 | 7 | 0.0085 | Cluster 3 |
| Toxin-Antitoxin | 64 | 143 | 0.0085 | Cluster 3 |
| Toxins | 41 | 73 | 0.00087 | Cluster 3 |

## 2.7. Comparison of CFU and RS ratio to baseline

The median CFU and RS ratio of each time point during the PAE phase were compared to the baseline (day 0) with calculated adjusted *p*-value (padj).

**Table F.** Recovery of CFU after 2 weeks of treatment

| Time | Median Log_10_ CFU | padj (Comparing timepoint to day 1) |
| --- | --- | --- |
| 1 | 5.86 | 1 |
| 5 | 6.09 | 0.80100176 |
| 7 | 5.93 | 0.91389221 |
| 11 | 5.77 | 0.80100176 |
| 14 | 5.85 | 0.80100176 |
| 21 | 5.81 | 0.80100176 |
| 28 | 6.13 | 0.90985585 |

**Table G.** Recovery of CFU after 4 weeks of treatment

| Time | Median Log_10_ CFU | padj (Comparing timepoint to day 1) |
| --- | --- | --- |
| 1 | 5.41 | 1 |
| 4 | 5.22 | 0.89515405 |
| 7 | 5.10 | 0.58715587 |
| 11 | 4.78 | 0.30197165 |
| 14 | 4.61 | 0.30197165 |
| 21 | 5.26 | 1 |
| 28 | 5.40 | 1 |

**Table H.** Recovery of RS ratio after 2 weeks of treatment

| Time | Median RS ratio | padj (Comparing timepoint to day 1) |
| --- | --- | --- |
| 1 | 20.52 | 1 |
| 5 | 47.48 | 0.00590896 |
| 7 | 48.91 | 0.00239611 |
| 11 | 57.97 | 0.00011954 |
| 14 | 61.20 | 0.00139673 |
| 21 | 69.67 | 0.00122635 |
| 28 | 78.57 | 0.00309677 |

**Table I.** Recovery of RS ratio after 4 weeks of treatment

| Time | Median RS ratio | padj (Comparing timepoint to day 1) |
| --- | --- | --- |
| 1 | 16.95 | 1 |
| 4 | 30.55 | 0.01542229 |
| 7 | 36.22 | 0.00456429 |
| 11 | 38.83 | 0.00456429 |
| 14 | 35.67 | 0.02057123 |
| 21 | 35.76 | 0.02109756 |
| 28 | 43.06 | 0.01623013 |

## 2.8. Transcriptional change of select gene sets during recovery in mice

**Fig E. Recovery of various processes in mice.** (a-p) Average of batch adjusted, VST-normalized, scaled gene expression in each treatment group over time for genes involved in (a) the TCA cycle, (b) NADH dehydrogenase Type I, (c) NADH dehydrogenase Type II, (d) Succinate dehydrogenase Type I and II, (e) ATPase, (f) aerobic respiration, (g) glycolysis, (h) glyoxylate bypass, (i) pentose phosphate pathway, (j) pyruvate dehydrogenase, (k) primary ribosomal proteins, (l) Antigen 85, (m) arabinogalactan synthesis, (n) fatty acid synthesis, (o) mycolic acid synthesis, and (p) peptidoglycan synthesis. Each point represents an individual sample, and the lines connect the mean for each time point. Values are centered around the average value for the pre-treated samples (gray line) so that points above and below zero represent upregulation and downregulation relative to pre-treated, respectively. (q) Hierarchical clustering of toxin genes (N=73) during the PAE phase. The heatmap shows the batch-adjusted, VST-normalized, scaled gene expression averaged across samples.

## 2.9. Table J. Toxin gene clustering in mouse samples

**Table J.** List of toxin genes sorted into each cluster in mouse samples

| Cluster | Included Toxins |
| --- | --- |
| Cluster 1 | Rv0065, Rv0299, Rv0301, Rv0582, Rv0598c, Rv0609, Rv0617, Rv0659c, Rv0910, Rv0960, Rv1102c, Rv1114, Rv1397c, Rv1546, Rv1982c, Rv2063A, Rv2494, Rv2527, Rv2602, Rv2829c, Rv3320c |
| Cluster 2 | Rv0240, Rv0595c, Rv0624, Rv0627, Rv0656c, Rv0749, Rv0836c, Rv0919, Rv1242, Rv1246c, Rv1495, Rv1561, Rv1720c, Rv1838c, Rv1942c, Rv1953, Rv1955, Rv1959c, Rv1962c, Rv1989c, Rv1991c, Rv2010, Rv2019, Rv2022c, Rv2103c, Rv2142c, Rv2231A, Rv2596, Rv2653c, Rv2697c, Rv2757c, Rv2759c, Rv2801c, Rv2863, Rv2866, Rv3180c, Rv3189, Rv3384c |
| Cluster 3 | Rv0059, Rv0549c, Rv0661c, Rv0665, Rv1741, Rv2530c, Rv2546, Rv2548, Rv2549c, Rv2872, Rv3182, Rv3358, Rv3408, Rv3749c |

# 3. References

1. Jiang, H., Lei, R., Ding, S.-W. & Zhu, S. Skewer: a fast and accurate adapter trimmer for next-generation sequencing paired-end reads. *BMC Bioinformatics* **15**, 182 (2014).

2. Staton, E. Pairfq. (2022).

3. Langmead, B., Trapnell, C., Pop, M. & Salzberg, S. L. Ultrafast and memory-efficient alignment of short DNA sequences to the human genome. *Genome Biol.* **10**, R25 (2009).

4. Putri, G. H., Anders, S., Pyl, P. T., Pimanda, J. E. & Zanini, F. Analysing high-throughput sequencing data in Python with HTSeq 2.0. *Bioinformatics* **38**, 2943–2945 (2022).

5. Cole, S. T. *et al.* Deciphering the biology of Mycobacterium tuberculosis from the complete genome sequence. *Nature* **396**, 190–190 (1998).

6. Lew, J. M., Kapopoulou, A., Jones, L. M. & Cole, S. T. TubercuList – 10 years after. *Tuberculosis* **91**, 1–7 (2011).

7. Soni, D. K., Dubey, S. K. & Bhatnagar, R. ATP-binding cassette (ABC) import systems of Mycobacterium tuberculosis: target for drug and vaccine development. *Emerg. Microbes Infect.* **9**, 207–220 (2020).

8. Kushwaha, A. K. & Bhushan, S. Unique structural features of the Mycobacterium ribosome. *Prog. Biophys. Mol. Biol.* **152**, 15–24 (2020).

9. Karbalaei Zadeh Babaki, M., Soleimanpour, S. & Rezaee, S. A. Antigen 85 complex as a powerful Mycobacterium tuberculosis immunogene: Biology, immune-pathogenicity, applications in diagnosis, and vaccine design. *Microb. Pathog.* **112**, 20–29 (2017).

10. Shao, Y. *et al.* TADB: a web-based resource for Type 2 toxin–antitoxin loci in bacteria and archaea. *Nucleic Acids Res.* **39**, D606–D611 (2011).

11. Abrahams, K. A. & Besra, G. S. Synthesis and recycling of the mycobacterial cell envelope. *Curr. Opin. Microbiol.* **60**, 58–65 (2021).

12. Schnappinger, D. *et al.* Transcriptional Adaptation of Mycobacterium tuberculosis within Macrophages: Insights into the Phagosomal Environment. *J. Exp. Med.* **198**, 693–704 (2003).

13. Kirksey, M. A. *et al.* Spontaneous phthiocerol dimycocerosate-deficient variants of Mycobacterium tuberculosis are susceptible to gamma interferon-mediated immunity. *Infect. Immun.* **79**, 2829–2838 (2011).

14. Pawełczyk, J. *et al.* Cholesterol-dependent transcriptome remodeling reveals new insight into the contribution of cholesterol to Mycobacterium tuberculosis pathogenesis. *Sci. Rep.* **11**, 12396 (2021).

15. Tallman, K. R., Levine, S. R. & Beatty, K. E. Small-Molecule Probes Reveal Esterases with Persistent Activity in Dormant and Reactivating Mycobacterium tuberculosis. *ACS Infect. Dis.* **2**, 936–944 (2016).

16. Lee, B. S., Sviriaeva, E. & Pethe, K. Targeting the cytochrome oxidases for drug development in mycobacteria. *Prog. Biophys. Mol. Biol.* **152**, 45–54 (2020).

17. Nobre, A., Alarico, S., Maranha, A., Mendes, V. & Empadinhas, N. The molecular biology of mycobacterial trehalose in the quest for advanced tuberculosis therapies. *Microbiology* **160**, 1547–1570 (2014).

18. Ditse, Z., Lamers, M. H. & Warner, D. F. DNA Replication in Mycobacterium tuberculosis. *Microbiol. Spectr.* **5**, (2017).

19. Voskuil, M. I. *et al.* Inhibition of Respiration by Nitric Oxide Induces a *Mycobacterium tuberculosis* Dormancy Program. *J. Exp. Med.* **198**, 705–713 (2003).

20. Remm, S., Earp, J. C., Dick, T., Dartois, V. & Seeger, M. A. Critical discussion on drug efflux in Mycobacterium tuberculosis. *FEMS Microbiol. Rev.* **46**, fuab050 (2021).

21. Rustad, T. R., Harrell, M. I., Liao, R. & Sherman, D. R. The Enduring Hypoxic Response of Mycobacterium tuberculosis. *PLOS ONE* **3**, e1502 (2008).

22. Gröschel, M. I., Sayes, F., Simeone, R., Majlessi, L. & Brosch, R. ESX secretion systems: mycobacterial evolution to counter host immunity. *Nat. Rev. Microbiol.* **14**, 677–691 (2016).

23. Duan, X., Xiang, X. & Xie, J. Crucial components of mycobacterium type II fatty acid biosynthesis (Fas-II) and their inhibitors. *FEMS Microbiol. Lett.* **360**, 87–99 (2014).

24. Slayden, R. A. & Barry, C. E. The role of KasA and KasB in the biosynthesis of meromycolic acids and isoniazid resistance in Mycobacterium tuberculosis. *Tuberc. Edinb. Scotl.* **82**, 149–160 (2002).

25. Wipperman, M. F., Sampson, N. S. & Thomas, S. T. Pathogen roid rage: cholesterol utilization by Mycobacterium tuberculosis. *Crit. Rev. Biochem. Mol. Biol.* **49**, 269–293 (2014).

26. Batt, S. M., Burke, C. E., Moorey, A. R. & Besra, G. S. Antibiotics and resistance: the two-sided coin of the mycobacterial cell wall. *Cell Surf. Amst. Neth.* **6**, 100044 (2020).

27. Domenech, P., Reed, M. B. & Barry, C. E. Contribution of the Mycobacterium tuberculosis MmpL protein family to virulence and drug resistance. *Infect. Immun.* **73**, 3492–3501 (2005).

28. Melly, G. & Purdy, G. E. MmpL Proteins in Physiology and Pathogenesis of M. tuberculosis. *Microorganisms* **7**, 70 (2019).

29. Quadri, L. E. N., Sello, J., Keating, T. A., Weinreb, P. H. & Walsh, C. T. Identification of a *Mycobacterium tuberculosis* gene cluster encoding the biosynthetic enzymes for assembly of the virulence-conferring siderophore mycobactin. *Chem. Biol.* **5**, 631–645 (1998).

30. Marrakchi, H., Lanéelle, M.-A. & Daffé, M. Mycolic Acids: Structures, Biosynthesis, and Beyond. *Chem. Biol.* **21**, 67–85 (2014).

31. Cook, G. M., Hards, K., Vilchèze, C., Hartman, T. & Berney, M. Energetics of Respiration and Oxidative Phosphorylation in Mycobacteria. *Microbiol. Spectr.* **2**, (2014).

32. Voskuil, M. I., Bartek, I., Visconti, K. & Schoolnik, G. K. The Response of Mycobacterium Tuberculosis to Reactive Oxygen and Nitrogen Species. *Front. Microbiol.* **2**, (2011).

33. Rens, C., Chao, J. D., Sexton, D. L., Tocheva, E. I. & Av-Gay, Y. Roles for phthiocerol dimycocerosate lipids in Mycobacterium tuberculosis pathogenesis. *Microbiol. Read. Engl.* **167**, (2021).

34. Maitra, A. *et al.* Cell wall peptidoglycan in Mycobacterium tuberculosis: An Achilles’ heel for the TB-causing pathogen. *FEMS Microbiol. Rev.* **43**, 548–575 (2019).

35. Raynaud, C. *et al.* Phospholipases C are involved in the virulence of Mycobacterium tuberculosis. *Mol. Microbiol.* **45**, 203–217 (2002).

36. Li, Y., Corro, J. H., Palmer, C. D. & Ojha, A. K. Progression from remodeling to hibernation of ribosomes in zinc-starved mycobacteria. *Proc. Natl. Acad. Sci.* **117**, 19528–19537 (2020).

37. Dahl, J. L. *et al.* The role of RelMtb-mediated adaptation to stationary phase in long-term persistence of Mycobacterium tuberculosis in mice. *Proc. Natl. Acad. Sci. U. S. A.* **100**, 10026–10031 (2003).

38. Hartman, T. *et al.* Succinate dehydrogenase is the regulator of respiration in Mycobacterium tuberculosis. *PLoS Pathog.* **10**, e1004510 (2014).

39. Thanna, S. & Sucheck, S. J. Targeting the trehalose utilization pathways of Mycobacterium tuberculosis. *MedChemComm* **7**, 69–85 (2016).

40. Maurya, R. K., Bharti, S. & Krishnan, M. Y. Triacylglycerols: Fuelling the Hibernating Mycobacterium tuberculosis. *Front. Cell. Infect. Microbiol.* **8**, 450 (2018).

41. Wan, T. *et al.* Structural insights into the functional divergence of WhiB-like proteins in *Mycobacterium tuberculosis*. *Mol. Cell* **81**, 2887-2900.e5 (2021).

42. Dow, A. *et al.* Zinc limitation triggers anticipatory adaptations in Mycobacterium tuberculosis. *PLoS Pathog.* **17**, e1009570 (2021).

43. Wynn, E. A. *et al.* Transcriptional adaptation of Mycobacterium tuberculosis that survives prolonged multi-drug treatment in mice. *mBio* **14**, e02363-23 (2023).
