# Supplementary material for: Physiologic recovery of Mycobacterium tuberculosis from drug injury: A molecular study of post antibiotic effect in vitro and in vivo
Source: PLoS Pathog. 2026 Apr 22;22(4):e1014131. doi: 10.1371/journal.ppat.1014131 (PMC13128115; doi:10.1371/journal.ppat.1014131)
Supplement: Attachment — Submitted filename: Response to Reviewers_2025.12.24.docx [file ppat.1014131.s004.docx]

**RESPONSE TO REVIEWERS**

We sincerely appreciate the close review and thoughtful critiques provided by the reviewers. We are pleased that reviewers found our work important, timely, and thought-provoking. The insightful reviewer comments led to major changes. Most importantly, we responded to the reviewers’ prompt to supplement our murine data by conducting a parallel *in vitro* evaluation of the physiologic changes that occur as *M. tuberculosis* recovers from drug exposure. The *in vitro* study constituted a major addition that substantially improved the manuscript. It reproduced key findings observed in the original mouse study in a way that was even more clear, thereby enhancing confidence in our conclusions.

**REVIEWER 1**

**The use of the RS ratio to look at the recovery of rRNA synthesis compared to overall transcriptional changes is a major strength of the work since this enables the authors to start to draw conclusions regarding the mechanism(s) that underlie the observed data. However this should be applied to in vitro PAE studies to provide a better connection between in vitro and in vivo experiments. This would then provide a framework for dissecting the contributions of the individual antibiotics to the PAE. Clearly measuring in vivo PAEs for individual drugs and different combinations would be a herculean task – the reviewer isn’t suggesting that this be done. However, as the authors point out, in vitro PAEs are normally measured by CFU counting. Thus a link between rRNA synthesis, physiological changes assessed by transcriptional response and CFU counting for in vitro PAEs of one or more drugs plus the combination would substantially strengthen the work.**

We are extremely grateful to the reviewer for this suggestion to include an in vitro PAE study and connect the in vivo and in vitro findings. We have fully embraced this idea and conducted an additional experiment examining Mtb recovery following HRZE exposure in vitro. We designed this experiment to determine if several key findings originally observed in mice were also seen *in vitro.* First, we wanted to know if physiologic changes during treatment and recovery were time dependent. *i.e.,* the longer the exposure the greater the injury and adaptation and the slower the recovery. Second, we wanted to know if the sequence of physiologic recovery observed in mice (rapid recovery of rRNA synthesis followed by slow transcriptional change with CFU as a lagging indictor) occurred *in vitro* as well. Third, we wanted to know if bacteria recovered fully in the absence of immune pressure present in the mouse model.

With these priorities in mind, in this new *in vitro* study, we evaluated two different exposure durations and monitored recovery to characterize the PAE phase more precisely.

We have modified all sections of the manuscript to incorporate and discuss these new in vitro findings (changes too extensive to provide line numbers here). We agree that this addition has significantly strengthened the manuscript by providing a clearer mechanistic link between molecular recovery and bacterial regrowth across both models.

We agree with the reviewer that an important next priority should be dissecting the contributions of individual antibiotics to treatment effects and PAE. We are keenly interested in how drugs contribute jointly to affect bacterial physiology during treatment and this work could be extended to the PAE period. A focus of our research program is using SEARCH-TB as a highly granular pharmacodynamic marker to more deeply understand the basis of drug interactions. As acknowledged by the reviewer, implementing our molecular approach for individual drugs and combinations to assess contribution to PAE would be a herculean task and the complexity of this project would likely overwhelm the current manuscript. We do absolutely agree with the suggestion and view this as an exciting avenue for future investigation.

Finally, we agree with the reviewer’s point that there is value in directly comparing PAE assessed in the traditional manner (*i.e.,* based in the lag in CFU counts) with PAE assessed with molecular measures of pathogen physiologic state (RS ratio & SEARCH-TB). This was not possible in mice because CFU did not rise during the 28-day observation period but it was possible *in vitro.* As highlighted above, this allowed us to describe the sequence of physiologic recovery relative to the traditional PAE measure.

**The reviewer recommends that the authors perform in vitro PAEs using RS ratio and transcriptional profiling in addition to CFU counting to provide a better link between in vitro and in vivo studies. This would provide additional insight into the mechanism(s) of the PAE. An experiment with the HRZE combination would be information that of course studies with individual drugs would be much better.**

In response to this excellent suggestion, we have incorporated new in vitro HRZE PAE experiments in which we evaluated Optical Density, CFU dynamics, RS ratio, and transcriptome changes over the recovery period. These additions have greatly enriched the manuscript and provide an important in vitro perspective that complements our in vivo observations. As noted above, we were not able to assess individual drug PAE.

**Additional background could be provided regarding the mechanism and importance of the PAE. For instance, that generation of a PAE is linked to dosing regimens (albeit for rapid growing bugs). In addition, there are actually several proposed mechanisms for the PAE that also include sequestration of antibiotic by the bacterium and that the antibiotic is still bound to the target. For instance, the relationship between the lifetime of the drug-target complex has been linked to target vulnerability. The authors could expand on proposed mechanisms and add appropriate references.**

We appreciate this prompt and now recognize that our single reference to “persistence of sublethal intracellular antibiotic” in the original submission was too obscure. To better contextualize our findings relative to hypotheses about PAE, we have added the paragraph below to the Discussion:

There are two general hypotheses about why PAE occurs: 1) slow recovery of cellular function as bacteria repair nonlethal damage and (2) intracellular persistence of the drugs (*i.e.,* non-dissociation from target).^48,50^ Our results are relevant to the first hypothesis. The time-dependent transcriptional change we observed during drug exposure is consistent with a progressive cascade of injury and adaptation. The time-dependent physiologic recovery we observed after drug exposure is consistent with slower recovery from “worse” injury. Because of the technical challenge of measuring non-dissociated drug, our design was unable to assess the alternative hypothesis that that PAE occurs because residual drug remains bound to its target within the bacterium even after extracellular concentrations have declined to undetectable levels. We note that intracellular persistence of drug is unlikely to explain the time-dependence of PAE since drug dissociation is understood to be a constant that should not change with exposure duration^51^ (*i.e.,* drug should not dissociate from its target more slowly after longer treatment than after shorter treatment). Critically, the cellular damage hypothesis and non-dissociated drug hypothesis are not mutually exclusive. Both could contribute to PAE in combination. Our results do not suggest that intracellular drug persistence is not also a factor.

**The investigators use a well-established drug cocktail for their experiments. Whilst this is relevant to the treatment of human disease, it would be interesting to know whether one or more of the drugs in the cocktail have a major effect on PAE. The authors state that studies of the in vivo PAE are more relevant than in vitro studies, however it would be useful to include in vitro data PAEs. For instance rifampin is known to generate a long in vitro PAE (70 h) whereas isoniazid generates a shorter PAE (18 h) and ethambutol a very short PAE (2 h). In contrast H+R is ~160 h.**

We agree with the importance of dissecting how individual drugs within the HRZE regimen interact to contribute to the overall PAE. As the reviewer noted, determining the PAE for each drug and combination—even *in vitro*—would be a herculean task, requiring multiple exposure and recovery experiments for each agent across different durations and concentrations. Analysis and summarization of transcriptome data from multiple drugs individually and in combinations would also be highly complex. While highly important, the scale seems beyond the scope of a single manuscript. We see evaluation of physiologic recovery after exposure to the full HRZE regimen as a ground-breaking first step that lays the groundwork for the project the reviewer has proposed.

**The observation that rRNA synthesis occurs well before the transcription of many other genes is a highly interesting observation. It would be interesting to know mechanistically why this is – is rRNA synthesis separate from other gene transcription events?**

We agree that it is highly interesting that rRNA synthesis occurs well before change in the transcriptome. This sequence was consistent between *in vitro* and murine drug exposures. Our study design does not provide mechanistic information about *why* recovery occurs in this sequence. We have emphasized the novelty and importance of this observation by adding the paragraph below.

A key observation in both *in vitro* and mouse experiments was sequential recovery of biologic processes. After the end of drug exposure, *Mtb* recovery began with resumption of rRNA synthesis, as indicated by a rapid increase in the RS ratio. Thereafter, there was a days-long pause before the emergence of meaningful transcriptional change. CFU was a lagging indicator, rising *in vitro* days after the transcriptome indicated recovery of metabolism and macromolecule synthesis. Although not previously reported in *Mtb* to our knowledge, a similar sequential order of recovery has been reported in *E. coli* and *B. subtilis,*^49^ starting with ribosome production before re-initiation of transcription of biosynthetic genes. The consistency of this finding from *in vitro* to mouse and across organisms suggests that there may exist a fundamental “order of operations" in the restoration of bacterial fitness.

**In addition what is the relationship between the physiological damage observed by transcriptional profiling and bacterial growth? This seems to be a critical gap in knowledge – e.g. how much ‘repair’ is needed before regrowth can commence?**

We thank the reviewer for this excellent question. We agree that repair is likely a progressive process in which there is a continuum of recovery (as implied by the reviewer’s “how much” question).

Correlating the bacterial transcriptome with bacterial replication is complicated by the likelihood that there may be a continuous spectrum of replication states ranging from non-replicating to slowly replicating to rapidly replicating. This slow “ramp-up” would make it hard to discern when regrowth commences. Additionally, CFU is an insensitive marker of the initiation of replication because the bacterial burden must increase substantially before a CFU increase is discernable. This inability to precisely quantify the replication state makes addressing the comment a challenge.

We think that this interesting question needs to be addressed in new experiments designed specifically to connect measures of bacterial physiology with accurate measures of bacterial growth such as time-lapse microscopy with microfluidics or the replication clock plasmid. To link physiology with replication, such an experiment would require granular sampling designed specifically to capture cellular changes during the time frame that replication is anticipated to start. Because our *in vitro* experiment was not designed for this purpose, our current data do not allow us to determine how much repair is required before regrowth can begin, but we fully agree that this represents an important direction for future study.

**Some might argue that the bacteria that regrow following drug exposure represent a ‘persister’ population. The authors might like to add some discussion regarding this point.**

We appreciate this prompt and have added the following paragraph to the Discussion.

Bacteria that tolerate drug exposure despite an absence of resistance-conferring mutations have been called “persisters.”^52^ The small minority *Mtb* population that withstood weeks of HRZE treatment in mice (2.8% and 1.0% of the original population after 2-weeks and 4-weeks, respectively) could be considered persisters. However, this work and our previous work with individual drug exposures in mice^42^ argue against a singular persister phenotype. Instead, we observe a time-dependent^8^ and drug-specific^42^ range of transcriptional injury and adaptation suggesting that there may be a spectrum of drug-tolerant states rather than a single a uniform persister phenotype.

**REVIEWER 2**

**RS ratios could be described a little more in methods and results/discussion. How are they determined and what do they actually mean? The authors refer back to their earlier work, but I could not find enough detail to think I’d be able to reproduce that work.**

We agree that it is crucial that readers understand the basis and meaning of the RS ratio. We have therefore revised the sentence below in the Introduction to clarify interpretation of RS ratio results.

The RS ratio quantifies *Mtb* rRNA synthesis based on detection of short-lived precursor rRNA sequence.^20^ A higher RS ratio indicates an active *Mtb* phenotype with higher ongoing rRNA synthesis whereas a low RS ratio indicates a quiescent *Mtb* phenotype with less ongoing rRNA synthesis.

We also substantially increased detail in the methods section about how the RS ratio is performed. For the Discussion, we focused on the inferences that can be drawn from RS ratio results which are the following. First, the RS ratio shows that both *in vitro* and in mice, physiologic recovery from drug exposure depends on the duration of drug exposure (*i.e.,* the longer the exposure, the slower the recovery). This is consistent CFU and SEARCH-TB results and these showing the same pattern with three independent measures across two systems (*in vitro* and mouse) enhances confidence. Second, the RS ratio illustrates the sequential nature of recovery, starting with resumption of rRNA synthesis, then mRNA change and finally CFU change. As discussed in response to Reviewer 1 above, this sequence of recovery was observed both *in vitro* and in mice.

**The week 2 and week 4 treatment groups are compared throughout the manuscript but there are no statistical analyses comparing then, can statistics be performed between these groups? For example, adding statistics to Fig 3b-l.**

We appreciate this prompt. In the course of our major revision to incorporate the *in vitro* experiments requested by reviewers, we remained cognizant of the request for formal statistical testing. We now provide the requested p-values for both *in vitro* and mouse results. We chose not to include p-values directly in Figures, as the number of comparisons would make the figure overly complex and potentially confusing.

**It would be good to see the variation between mouse replicates within each treatment group. How does that variation compare to the differences between week 2 and week 4? Can differences between week 2 and week 4 groups be explained by variation between mouse replicates?**

We thank the reviewer for this constructive comment. We have changed the format of our expression plots to replace error bars with dots representing individual mouse results. This is a more transparent and effective way to allow readers to assess variation between mouse replicates. The overall spread generally appears comparable between treatment groups, indicating similar variation among biological replicates. We do not find evidence that differences between weeks 2 and 4 are explicable based on differences in variation. As with the previous comment, we remained aware of this request as we added large sections on the *in vitro* results and also show individual *in vitro* replicates rather than error bars.

**Line 214: Acronyms BLQ and LOQ are used but not described.**

Thank you for bringing this omission to our attention. We have spelled out the acronym LOQ as “Limit of Quantification” and have replaced BLQ with “below LOQ.”

**Can the authors better define the expected transcriptome of bacteria experiencing post-antibiotic effect (PAE) and if their results match what is expected? We might for example imagine that proteins and/or DNA become damaged and need repair. Are proteases/DNA repair enzymes/oxidative stress response genes differentially regulated?**

To our knowledge, the transcriptome of Mtb *specifically* during the PAE phase has not previously been defined. We therefore did not have a reference transcriptome for comparison.

Nonetheless, transcriptional change during recovery does seem broadly consistent with our expectations. Our previous work elucidated the pattern of injury and adaptation induced by HRZE exposure. We anticipated that these transcriptional changes would gradually be reversed during recovery. This is indeed what occurred *in vitro* as the bacterium recovered to be nearly indistinguishable from control. In mice, we observed only partial recovery of the transcriptome which seems plausibly attributable to the presence of adaptive immunity.

SEARCH-TB presents an “embarrassment of riches” in the sense that there are vastly more physiologically important changes than can be represented in text. We hope the accompanying online analysis tool will enable readers to explore the biological processes that they are interested in. We do appreciate the prompt to summarize changes in specific processes that relate to repair. Figures 3 and 6 now include subfigures showing that (1) expression of genes for the ClpP1P2 protease complex that is essential to proteostasis significantly rose during recovery, (2) expression of genes for DNA gyrase needed for DNA repair and replication rose during recovery and (3) expression of genes involved in oxidative stress response had increased expression early in recovery which then declined.

**Can the authors include a supplemental file of all their curated gene sets and the list of genes in each set? This would help other researchers!**

We are glad to hear that the reviewer considers this useful and would be happy to make it accessible to other researchers. Therefore, we have made Supplemental file 2 with all the gene categories we consider, and the genes included within each category. This information is also available via the online analysis tool: https://microbialmetrics.org/analysis-tools/.

**Fig S2: Can the authors explain why there are significant differences between the two experiments, and why the relationship is not linear with the more highly expressed genes?**

We appreciate the careful review. To clarify, Fig S2 in the submission (now labeled as Fig S4) plots the fold-change of all 2568 genes relative to control in the 2019 experiment with fold-change relative to control in the 2023 experiment. Positive and negative numbers mean increased or decreased expression relative to control at weeks 2 or 4. Since a gene with lower expression could have a large fold-change, this plot does not provide information about whether a gene had higher or lower expression.

To us, the take-away was that the sets of genes that were either up- or down-regulated after HRZE exposure were consistent in two independent experiments. We do note that the current experiment did have larger fold-changes than observed in the original Wynn manuscript. Since we have verified the reproducibility of the SEARCH-TB platform, we think that these between-experiment differences in fold-change magnitude is probably biological rather than technical. The two mouse experiments with HRZE were conducted in the same laboratory (Robertson Lab, Colorado State University) but with a 4-year gap between studies. Since the differentially expressed genes are similar, the larger scale of fold-change in the second experiment is unlikely to alter the central biologic conclusions drawn.

**The authors structure their manuscript around “treatment forgiveness” and the physiological changes that happen to bacteria in vivo when drug treatment is stopped. However, they do not present a transcriptome from Mtb that recover after treatment interruption or show how long the PAE lasts. It may be better to decrease focus on treatment forgiveness.**

We agree that it is appropriate to reduce emphasis on “forgiveness.” We have now eliminated reference to forgiveness from the Abstract, Introduction and Discussion.

**EDITOR**

**We do not publish any copyright or trademark symbols that usually accompany proprietary names, eg ©,  ®, or TM  (e.g. next to drug or reagent names). Therefore please remove all instances of trademark/copyright symbols throughout the text, including:
- ® on pages: 2, and 5.**

In accordance with journal policy, we have removed two instances of the trademark symbols that are associated with the RS ratio.

**We have noticed that you have uploaded Supporting Information files, but you have not included a list of legends. Please add a full list of legends for your Supporting Information files after the references list.**

We have added a section after the reference list that provides a legend for our Supporting Information.

**Some material included in your submission may be copyrighted.**

We believe the concern about potentially copyrighted material in our original Figure 1 may have been the mouse icons. We have removed the mouse icons from Figure 1 and use colored dots instead.

**Thank you for stating "Sequence data are available via accession number PRJNA1140268." Please note that, though access restrictions are acceptable now, your entire minimal dataset will need to be made freely accessible if your manuscript is accepted for publication.**

We confirm that our dataset is set to automatically become publicly available upon publication of the manuscript.

**Please amend your detailed Financial Disclosure statement. This is published with the article. It must therefore be completed in full sentences and contain the exact wording you wish to be published.**

1. **State what role the funders took in the study. If the funders had no role in your study, please state: "The funders had no role in study design, data collection and analysis, decision to publish, or preparation of the manuscript."
   If any authors received a salary from any of your funders, please state which authors and which funders.**

We have added the comment “the funders had no role in study design, data collection and analysis, decision to publish or preparation of the manuscript.”

**Please ensure that the funders and grant numbers match between the Financial Disclosure field and the Funding Information tab in your submission form. Note that the funders must be provided in the same order in both places as well. Currently, the order of the grants is different in both places.**

We have verified that the grant numbers match between the Financial Disclosure field and the Funding Information tab.

**Please provide a completed 'Competing Interests' statement, including any COIs declared by your co-authors. If you have no competing interests to declare, please state "The authors have declared that no competing interests exist".**

We have added the statement “The authors have declared that no competing interest exist.”
