# Supplementary material for: Physiologic recovery of Mycobacterium tuberculosis from drug injury: A molecular study of post antibiotic effect in vitro and in vivo
Source: PLoS Pathog. 2026 Apr 22;22(4):e1014131. doi: 10.1371/journal.ppat.1014131 (PMC13128115; doi:10.1371/journal.ppat.1014131)
Supplement: Attachment — Submitted filename: Response to Reviewers2_2026.01.22.docx [file ppat.1014131.s005.docx]

**RESPONSE TO REVIEWERS**

We are pleased that the reviewer found our revision to be a significant improvement. We are again grateful that the reviewers led us to perform *in vitro* studies which are now inspiring further follow-on questions and proposals. The reviewer’s expertise on drug-target retention has led us to more precisely articulate the role of retention in PAE. We hope that this is now accurate and clear.

**REVIEWER 1**

**Regarding this point: “There are two general hypotheses about why PAE occurs: 1) slow recovery of cellular function as bacteria repair nonlethal damage and (2) intracellular persistence of the drugs (i.e., non-dissociation from target).48,50”**

**Intracellular persistence of drug is normally used to refer to a general scenario where the drug remains in the cell. This can occur through different mechanisms. For instance, the drug could be trapped in the cell (slow efflux) or nonspecifically bound to cellular structures. In my review I was referring to a specific mechanism in which the drug is still bound to the drug target because the drug has a long residence time on the target.**

**In all these scenarios the drug will remain bound to the target when the extracellular drug concentration has decreased (e.g. in the washout step of a PAE). However, only in the situation where the drug has a long residence time on the target (slow off rate or covalent) will non-dissociation from the target actually be the reason for drug still being in the cell which as implied by the authors.**

**I think this is an important distinction between the general phenomenon of intracellular persistence of the drug and the lifetime of the drug target complex, which I encourage the authors to make in their manuscript and add appropriate references. For instance, there are examples where the PAE has been explicitly linked to the slow off rate of the drug from the target. This is the proposed mechanism for the long PAE of ganfeborole in Mtb (doi:10.1021/acschembio.5c00705). There are also other examples – e.g. the ribosome and LpxC).**

**Of course, whether or not long drug-target residence time translates to a PAE depends on target vulnerability and the rate of target synthesis (e.g. covalent b-lactam inhibitors of PBPs do not generate a PAE in Gram negatives). This was the other point I was making in my review.**

We absolutely appreciate this guidance. We have revised the relevant section of the Discussion (lines 669-690) in a way that we hope more accurately captures the state of the literature.

**Secondly, there are a number of articles that refer to the observation that in vivo PAEs are often longer than in vitro PAES – this could be due to immune pressure, nutrient limitation that alters target vulnerability or possibly trapping/accumulation of drug in the infected tissue. Thus, I am not sure that it is warranted to use the word ‘novel’ in this context: “…novel finding that PAE is longer in mice than in vitro suggests that immunity may augment PAE” I acknowledge that most in vivo studies are for rapid growing bacteria. It is of course very difficult to know what the local drug concentration is in the infected tissue but it is possible that the tissue acts as a reservoir that clears more slowly. PET imaging or ex vivo mass spec imaging are ways to do this (albeit non trivial).**

Indeed, others have previously shown that PAE is longer *in vivo* than *in vitro* for other bacteria. Our finding that this is also true for *M. tuberculosis* represents an extension of existing findings rather than a novel finding. We have therefore removed the emphasis on novelty from the final sentences of the Abstract. We have retained the section of the Discussion (lines 691-695) that discuss previous evidence from other bacteria that PAE is longer *in vivo.*
